# Unscrambling Egg Allergy: The Diagnostic Value of Specific IgE Concentrations and Skin Prick Tests for Ovomucoid and Egg White in the Management of Children with Hen's Egg Allergy

**DOI:** 10.5402/2012/627545

**Published:** 2012-02-09

**Authors:** D. E. Marriage, M. Erlewyn-Lajeunesse, D. J. Unsworth, A. J. Henderson

**Affiliations:** ^1^Clinical Investigation Unit, Bristol Royal Hospital for Children, Upper Maudlin Street, Bristol BS2 8BJ, UK; ^2^Paediatric Allergy, Immunology and Infectious Diseases, Southampton University Hospital NHS Trust, Tremona Road, Southampton SO16 6YD, UK; ^3^Immunology Department, Southmead Hospital, Bristol BS10 5NB, UK; ^4^School of Social and Community Medicine, University of Bristol, Oakfield House, Oakfield Grove, Bristol BS8 2BN, UK

## Abstract

Resolution of egg allergy occurs in the majority of egg allergic children. Positive specific IgE antibodies to ovomucoid (OVM) have been suggested to be of greater predictive value for persistent egg allergy than specific IgE to egg white. The performance of OVM-specific IgE antibody levels in a cohort of children referred for a routine egg challenge was compared with egg white specific IgE levels in predicting a positive egg challenge. 24/47 subjects had persistent egg allergy. Receiver operating characteristic analysis showed that OVM-specific IgE testing was the most useful test for the diagnosis of persistent egg allergy. The optimal decision points for the prediction of persistent egg allergy were >0.35 kU_A_/L for specific IgE levels to both EW and OVM, and ≥3 mm for SPT. Children with specific IgE levels suggestive of persistent egg allergy need not be subject to an egg provocation challenge, reducing both costs and risks to the child.

## 1. Introduction

Egg allergy is common in early childhood, affecting 1-2% of all preschool children and may be associated with severe symptoms, including anaphylaxis [1–3]. Although remission of type 1 hypersensitivity to hen egg occurs in the majority of cases, hypersensitivity may persist through adolescence into adulthood, in which 12% of food allergies are attributed to egg [4–9]. Resolution of egg allergy may first manifest with tolerance to cooked egg products despite continued reaction to raw egg, whilst in others, allergy may persist to egg in any form [10–12]. The ubiquitous inclusion of egg in prepared foods as a result of its useful functional properties—including binding and emulsifying—renders complete egg avoidance difficult. The sooner tolerance to egg is ascertained, the sooner a child can enjoy a normal, unrestricted diet. This alleviates the social and emotional burden associated with having a food allergy and is important for normal growth and development [13]. Children with any form of egg allergy also need to be identified for vaccination purposes as both seasonal and pandemic influenza vaccines and yellow fever vaccine are contraindicated.

The gold standard for the diagnosis of egg allergy is the double-blind placebo-controlled food challenge (DBPCFC) which is both resource and time expensive, and potentially hazardous [10, 14]. Routine skin prick and specific IgE antibody tests to whole egg white are of limited value and have poor sensitivity for the identification of children with allergy to raw egg. Many potentially allergenic egg proteins exist; the major recognized allergens being ovomucoid (Gal d 1), ovalbumin (Gal d 2), ovotransferrin (Gal d 3), and lysozyme (Gal d 4) [15–17]. Uniquely among these, OVM retains its antibody-binding activity despite extensive heating of up to an hour at 100°C and is stable against digestion by proteinases [10, 15, 17–19]. This characteristic may have utility in the diagnosis of persistent egg allergy. Järvinen and colleagues reported that specific IgE antibodies to sequential epitopes of ovomucoid were associated with the persistence of egg allergy [20]. As OVM retains it allergenic properties despite extensive heating, OVM-specific IgE is suggested to be of value in the identification of children with allergy to both cooked and raw form of egg. Previous studies have also clearly identified the value of ovomucoid-specific IgE levels in children with allergy specifically to extensively heated egg [4]. This study has been designed to evaluate the usefulness of measuring specific IgE to OVM as a screening test for those children in whom an oral provocation challenge is being considered.

## 2. Materials and Methods

### 2.1. Study Design

Children (2–16 years old) attending the Paediatric Allergy Clinics at Bristol Royal Hospital for Children from July 2009 to February 2010 with egg allergy previously confirmed by positive history and positive SPT or specific IgE to hen egg and clinically suspected of having outgrown their egg allergy were recruited. Children with an inconclusive clinical history were excluded as were those with the absence of previously documented specific IgE to egg white or positive SPT. The study protocol was approved by the Ethics Committee of North Somerset and South Bristol, UK. Written informed consent was obtained from the parent of each subject.

**Figure 1:**
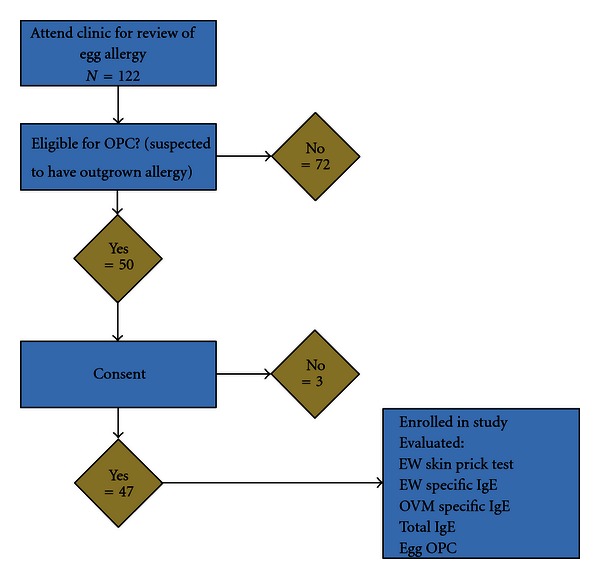
Pathway of subjects through the study.

Serum concentrations of specific IgE antibodies (sIgE) to OVM and egg white, and total IgE were measured at the clinic visit. Skin prick test responses to egg white were also measured. All eligible children subsequently had an oral egg provocation challenge (OPC).  Figure 1 shows the pathway of subjects through the study protocol.

### 2.2. Specific IgE Antibody Testing

Specific IgE concentrations in venous blood to egg white (EW) and ovomucoid (OVM) were measured using ImmunoCap (Phadia AB, Uppsala, Sweden).

### 2.3. Skin Prick Testing

Allergen skin prick tests were performed by a single, trained paediatric allergy nurse using a commercial one-prick lancet technique on the volar aspect of the forearm. Commercially available extracts of egg white using a 1 : 20 (wt/vol) solution (Soluprick, ALK, Uppsala, Sweden) were used with histamine dihydrochloride (10 mg/mL, ALK-Abello A/S, Horsholm, Denmark) as a positive control and saline (Soluprick SQ, ALK-Abello) as a negative control. The maximal skin wheal diameter (mm) was measured after 15 minutes.

Based on the OPC, children who reacted were classified as having persistent egg allergy and children tolerant of egg throughout the challenge were classified as having resolved egg allergy.

### 2.4. Statistics

An SPT reaction was considered positive if the resultant wheal was >3 mm in diameter in the presence of a reaction to histamine of at least 3 mm in diameter and a negative response to the corresponding negative control. Specific IgE antibody levels ≥0.35 kU_A_/L were considered positive according to the manufacturer's instructions.

The likelihood of a positive OPC following a positive reaction in each of three screening tests: SPT to egg white; raised specific IgE levels to egg white; raised specific IgE levels to ovomucoid, were compared. Fagan's nomogram was used to calculate post-test probability [21]. Univariate logistic regression analyses of persistent egg allergy as confirmed by positive OPC by each screening test were performed. Comparisons of non-normally distributed continuous variables used ANOVA tests or Mann-Whitney *U* test. Dichotomous variables were analysed using the *χ* ^2^ test. 2 by 2 contingency tables were used to calculate sensitivity and specificity for SPT and specific IgE cut-off levels and receiver operator characteristic (ROC) curves were constructed. For all tests a *P* value of < 0.05 was considered to be statistically significant. All calculations were carried out with the statistical analysis program PASW Statistics 18.0 (Chicago, IL).

## 3. Results

Forty-seven children, aged 2–16 years (median 4.6 years; 19 girls) were recruited and completed an egg OPC. All children had been under annual review in the allergy clinic following their initial presentation and had remained on an egg-free diet since diagnosis. Twenty-four children (51%) had a positive challenge; 10 (42%) reacted to extensively heated egg, 13 (54%) tolerated extensively heated egg but not freeze-dried powdered egg, and 1 reacted to raw egg. Twenty-three children with no response to OPC were classed as resolved food allergy.

### 3.1. Screening Tests

**Figure 2:**
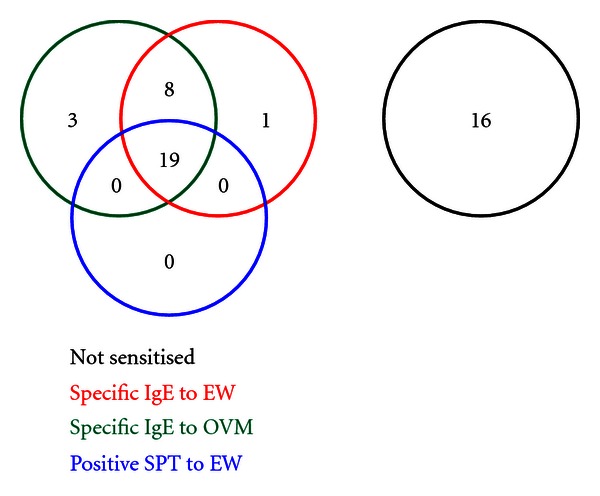
Sensitisation to hen's egg white.

**Figure 3:**
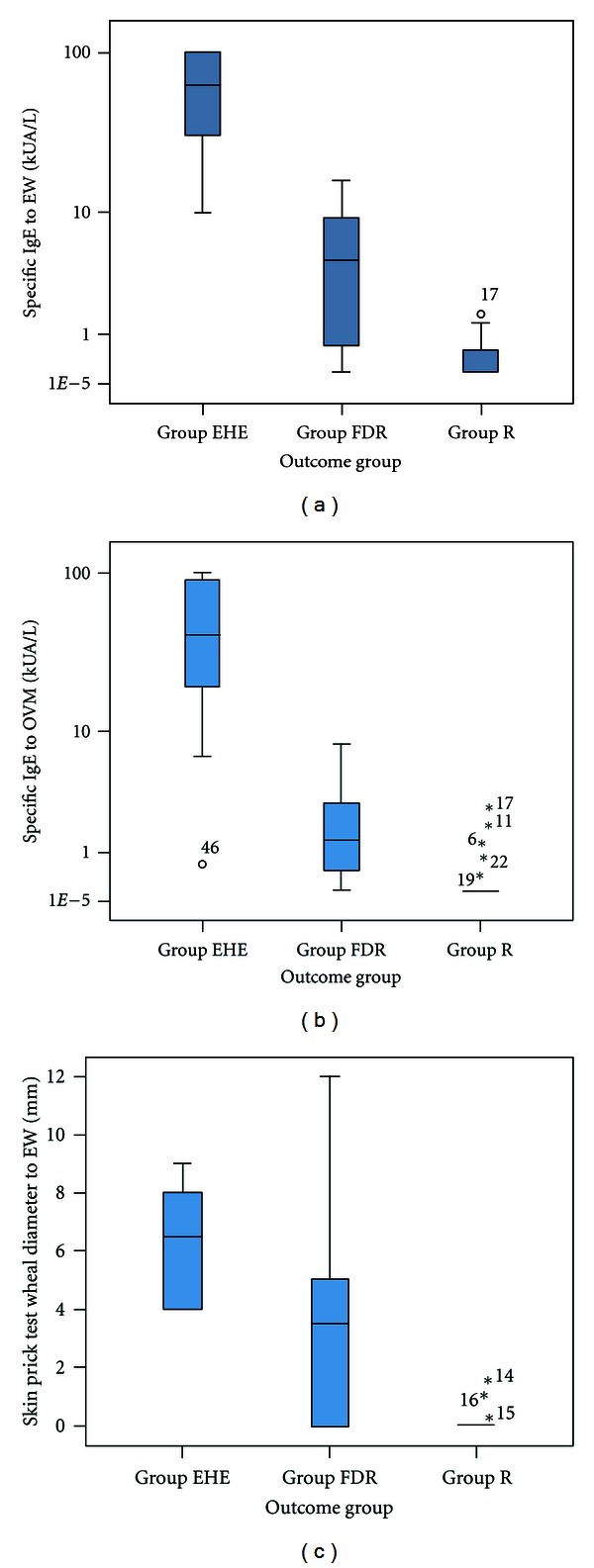
EW SPT and specific IgE levels and OVM-specific IgE levels in children with extensively heated egg allergy (EHE), freeze-dried, or raw egg allergy (FDR) and resolved egg allergy (R).

Figure 2 shows that of the 47 participating children, of the 47 participating children, 31 (66%) were sensitised to hen's egg at screening (positive SPT or specific IgE to egg white and/or ovomucoid). 17 (36%) of these had a positive SPT to EW; mean wheal diameter 6.5 mm (median [range], 5.5 mm [3–12 mm]). Each of these 17 children also had detectable specific IgE to EW. 30 children (64%) had detectable EW-specific IgE (median kU_A_/L [range], 6.4 [0.40–101]). 28 children (60%) had detectable OVM-specific IgE (median kU_A_/L [range], 2.2 [0.45–101]). 1 child (2%) was sensitised to OVM but had a negative specific IgE antibody level to EW. Three children (6%) with detectable specific IgE to EW were not sensitised to OVM.  Figure 3 shows area under the curve calculations for SPT to EW, and specific IgE levels to EW and OVM in predicting persistent egg allergy.

Univariate logistic regression showed an odds ratio for a positive egg OPC following a positive SPT as 2.7 (CI: 1.50–4.81), following a positive sIgE level for egg white was 3.3 (CI: 0.89–12.26) and following a positive sIgE level for ovomucoid as 7.4 (CI: 1.61–34.00).

### 3.2. ROC Curve Analysis of Three Tests for the Prediction of Allergy to Any Form of Egg

**Figure 4:**
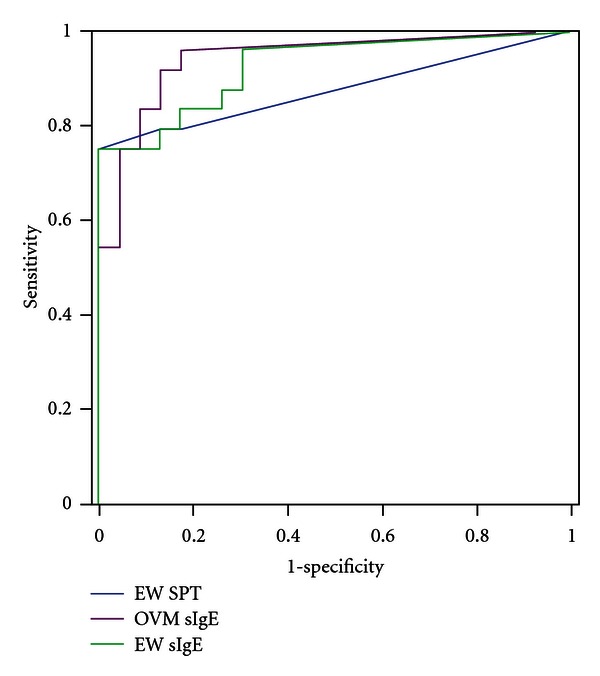
Receiver-operator characteristic curves showing the performance of the three screening tests in children with persistent and resolved egg allergy in predicting any form of egg allergy.

Figure 4 shows the ROC curves for SPT to egg white, and specific IgE concentration tests for egg white and ovomucoid, in predicting children with allergy to any form of egg. The ROC test statistics for each of the predictor variables are shown. The area under the curve was greater for ovomucoid sIgE (0.94 (95% CI) than for egg white SPT or sIgE but these differences were not significant. Optimal cut-off values useful for defining children likely to have persistent egg allergy were ≥3 mm for EW-SPT and ≥0.35 kU_A_/L for specific IgE levels for EW and OVM. Analysis of the diagnostic utility of the three tests in predicting reactions to extensively heated egg is available (Table 1).

## 4. Discussion

This study showed a SPT wheal diameter ≥3 mm to egg white to be very highly predictive of persistent egg allergy, although it could not be used to distinguish children who could tolerate extensively heated eggs from those who could not. However, skin test specificity was poor with 6 children (25%) with persistent allergy having a negative SPT result. A positive specific IgE level to ovomucoid had a greater positive predictive value and increased post-test probability of persistent egg allergy than specific IgE to egg white. Children who could not tolerate cooked egg had significantly higher specific IgE levels to both egg white and ovomucoid than children with resolved egg allergy or those able to tolerate extensively heated egg.

The SPT results in this study concur with the work of Sampson and Ho who reported a 3 mm SPT wheal diameter to have high sensitivity but a low specificity and therefore to be of limited clinical value. Diagnostic specific IgE cut-off levels in this study have been reported using 95% positive predictive values to establish decision points [22, 23]. Other studies have used the 95% specificity of a screening test as this is not dependent upon disease prevalence and can be extrapolated between a tertiary allergy clinic and other relevant clinical settings [14]. However, the published cut-off values which are predictive of clinical reactivity with greater than 95% certainty vary considerably between studies. For predicting egg allergy Boyano-Martínez et al. published a 95% specific cut-off value of 0.35 kU_A_/L, Osterballe and Bindslev-Jensen reported a value of 1.5, Sampson et al. reported 6 kU_A_/L, Ando reported 7 kU_A_/L, Celik-Bilgili et al. reported 10 kU_A_/L and Komata et al. reported a cut-off value of 25.5 kU_A_/L [14, 22, 24–27]. Within this tertiary clinic population the optimal 95% specific cut-off values for specific IgE levels to egg white and ovomucoid were both 0.35 kU/L.

Published cut-off specific IgE levels for ovomucoid, as for egg white, vary considerably between the few available studies. Cut-off values are higher in published studies than reported in this paper. Ando et al. reported that a cut-off value of 11 kU_A_/L was predictive of extensively heated egg allergy whilst a value of 7 kU_A_/L was predictive of raw egg allergy only, whilst Lemon-Mulé reported a cut-off value of 50 kU_A_/L to be 90% predictive of heated egg allergy [14, 28].

Lemon-Mulé and co-workers found regular ingestion of heated egg in egg allergic children was associated with immunological changes including a reduction in skin prick test wheal diameters to egg white. Specifically, continued ingestion of heated egg was associated with a reduction in ovalbumin-specific IgE levels and an increase in ovalbumin-specific and ovomucoid-specific IgG4 levels [28]. 

Therapeutic strategies for food allergy are currently of great interest, with particular focus upon oral desensitisation to foods. Recent evidence suggests that diets containing extensively heated egg may be an effective method of oral desensitisation, making clarification of each child's egg allergic status of increased importance [19]. It is also possible that children who will never lose their egg allergy still become tolerant of extensively heated egg allergy. Additionally, having a less-restricted diet makes life easier for these children and their families, so knowledge of their degree of egg tolerance remains socially as well as clinically useful.

The strengths of this study lie in the well-characterised clinic population. All food challenges were performed using the same protocol and supervised by one individual for consistency. SPT was performed by a single trained clinical nurse specialists reducing interoperator variability. A limitation of this study was the difference in composition of the two subgroups for children with persistent egg allergy, which makes the data difficult to compare with previously published studies. Most previous studies report a large number of children with raw egg allergy, which were not observed in this study. Additionally many studies have focused on atopic prognostic factors which have not been examined closely in this study. Factors of potential interest, including information on the number of previous reactions, were also not recorded. This study only included children over the age of 2 years due to recognised differences in specific IgE levels and SPT wheal diameters in infants and children to enable more precise data analysis, and all children had previous confirmation of their egg allergy rather than just positive IgE results. This varies from some previous studies where definitive confirmation has not been required.

This study has shown a clear difference in median specific ovomucoid IgE levels between children with extensively heated egg allergy and children only reacting to freeze-dried or raw egg. The optimal decision points for predicting that a child would have an allergic reaction to extensively heated egg on OPC with at least 95% certainty were 10 kU_A_/L for egg white and 6 kU_A_/L for ovomucoid. In opposition to the work of Ando et al., specific IgE levels to OVM were not superior in the diagnosis of allergy to EHE than specific IgE levels to EW. Three of the children with extensively heated egg allergy had higher specific IgE levels to OVM than EW. This may be of clinical significance in predicting allergy to extensively heated egg and further research is justified to establish this distinction in a larger population.

## 5. Conclusions

In a group of children from a tertiary allergic clinic, measurement of specific IgE antibodies for ovomucoid was the best test for the prediction of persistent egg allergy. The use of skin testing as a basis for deciding to perform an OPC is limited without the additional information provided by specific IgE testing. The recommendations of this study are that specific IgE to ovomucoid should be used as an allergen marker prior to referring a child for an egg OPC test. However, despite improved screening, there remain a number of children in whom it is not possible to know whether a child has outgrown their egg allergy who will continue to need to be challenged in a safe environment.

## Figures and Tables

**Table 1:** 

Test	Area under the curve (95% confidence interval)	Sensitivity	Specificity
SPT egg white	0.88 (0.77–0.99)	75%	100%
sIgE egg white	0.92 (0.85–1.00)	96%	70%
sIgE ovomucoid	0.94 (0.85–1.00)	96%	78%
